# Metabolomics in Severe Aortic Stenosis Reveals Intermediates of Nitric Oxide Synthesis as Most Distinctive Markers

**DOI:** 10.3390/ijms22073569

**Published:** 2021-03-30

**Authors:** Beau Olivier van Driel, Maike Schuldt, Sila Algül, Evgeni Levin, Ahmet Güclü, Tjeerd Germans, Albert C. van Rossum, Jiayi Pei, Magdalena Harakalova, Annette Baas, Judith J. M. Jans, Jolanda van der Velden

**Affiliations:** 1 Amsterdam UMC, Vrije Universiteit Amsterdam, Physiology, Amsterdam Cardiovascular Sciences, De Boelelaan 1117, 1081 HZ Amsterdam, The Netherlands; m.schuldt@amsterdamumc.nl (M.S.); s.algul@amsterdamumc.nl (S.A.); a.guclu@isala.nl (A.G.); j.vandervelden1@amsterdamumc.nl (J.v.d.V.); 2 Amsterdam UMC, Universiteit van Amsterdam, Internal and Vascular Medicine, Meibergdreef 9, 1105 AZ Amsterdam, The Netherlands; e.levin@amsterdamumc.nl; 3 Amsterdam UMC, Vrije Universiteit Amsterdam, Cardiology, Amsterdam Cardiovascular Sciences, De Boelelaan 1118, 1081 HV Amsterdam, The Netherlands; t.germans@amsterdamumc.nl (T.G.); ac.vrossum@amsterdamumc.nl (A.C.v.R.); 4 University Medical Center Utrecht, Universiteit Utrecht, Cardiology, Heidelberglaan 100, 3584 CX Utrecht, The Netherlands; J.Pei-2@umcutrecht.nl (J.P.); M.Harakalova-2@umcutrecht.nl (M.H.); 5 University Medical Center Utrecht, Universiteit Utrecht, Genetics, Heidelberglaan 100, 3584 CX Utrecht, The Netherlands; a.f.baas@umcutrecht.nl (A.B.); j.j.m.jans@umcutrecht.nl (J.J.M.J.)

**Keywords:** aortic stenosis, metabolomics, biomarker, metabolic profile

## Abstract

Background: Calcific aortic valve disease (CAVD) is a rapidly growing global health problem with an estimated 12.6 million cases globally in 2017 and a 112% increase of deaths since 1990 due to aging and population growth. CAVD may develop into aortic stenosis (AS) by progressive narrowing of the aortic valve. AS is underdiagnosed, and if treatment by aortic valve replacement (AVR) is delayed, this leads to poor recovery of cardiac function, absence of symptomatic improvement and marked increase of mortality. Considering the current limitations to define the stage of AS-induced cardiac remodeling, there is need for a novel method to aid in the diagnosis of AS and timing of intervention, which may be found in metabolomics profiling of patients. Methods: Serum samples of nine healthy controls and 10 AS patients before and after AVR were analyzed by untargeted mass spectrometry. Multivariate modeling was performed to determine a metabolic profile of 30 serum metabolites which distinguishes AS patients from controls. Human cardiac microvascular endothelial cells (CMECs) were incubated with serum of the AS patients and then stained for ICAM-1 with Western Blot to analyze the effect of AS patient serum on endothelial cell activation. Results: The top 30 metabolic profile strongly distinguishes AS patients from healthy controls and includes 17 metabolites related to nitric oxide metabolism and 12 metabolites related to inflammation, in line with the known pathomechanism for calcific aortic valve disease. Nine metabolites correlate strongly with left ventricular mass, of which three show reversal back to control values after AVR. Western blot analysis of CMECs incubated with AS patient sera shows a significant reduction (14%) in ICAM-1 in AS samples taken after AVR compared to AS patient sera before AVR. Conclusion: Our study defined a top 30 metabolic profile with biological and clinical relevance, which may be used as blood biomarker to identify AS patients in need of cardiac surgery. Future studies are warranted in patients with mild-to-moderate AS to determine if these metabolites reflect disease severity and can be used to identify AS patients in need of cardiac surgery.

## 1. Introduction

Calcific aortic valve disease (CAVD) is a rapidly growing global health problem with an estimated 12.6 million cases globally in 2017 and a 112% increase of deaths since 1990 due to aging and population growth [1]. CAVD may develop into aortic stenosis (AS) by progressive narrowing of the aortic valve. Calcification of the aortic valve develops through a process similar to atherosclerosis, which starts with endothelial damage followed by inflammation, fibrosis and calcification of the valve [2]. The subsequent narrowing of the aortic valve results in a gradual obstruction of the left ventricular (LV) outflow tract and increased afterload, leading to compensatory remodeling characterized by LV hypertrophy (LVH) to preserve cardiac output. During this compensatory phase AS can be asymptomatic for multiple years, hiding a pathological process which, once symptomatic, has a poor prognosis if left untreated [3]. Once AS becomes symptomatic, the LV is no longer able to generate the force needed to maintain flow through the narrowed aortic valve and produce sufficient cardiac output. In this advanced disease stage reverse remodeling starts, characterized by irreversible myocardial fibrosis and myocyte death due to myocardial ischemia, ultimately leading to heart failure [4]. The transition from compensatory to adverse LV remodeling and heart failure can be prevented by timely aortic valve surgery to correct AS-induced pressure overload. Blood biomarkers, in combination with imaging-based cardiac characterization, may aid in defining the optimal timing of aortic valve surgery.

Heart failure is accompanied by significant changes in myocardial energetics and metabolic homeostasis. The failing myocardium is energy deficient, characterized by a reduction of myocardial high-energy phosphate stores as shown in multiple preclinical and clinical studies [5,6,7,8]. Myocardial energy efficiency, measured as the ratio between cardiac external work and oxygen consumption, is reduced in AS and is an important determinant for functional outcome of aortic valve replacement [9,10]. Metabolic changes in heart failure include a shift of substrate metabolism from preferred free fatty acids (FFA) to glucose to generate ATP in a more oxygen-efficient manner [11]. The shift from FFA to glucose has been observed even before the onset of heart failure in mice with pressure-overloaded cardiac hypertrophy as a result of abdominal aortic constriction [12]. Metabolic profiling may thus aid in staging LV disease severity in patients with AS.

The human heart has an immense metabolic demand of 30 g of fat and 20 g carbohydrates per day and its metabolic flexibility allows it to consume nearly all types of energy substrates in order to form ATP [13], determined by external factors such as the availability of substrates in the blood [11] or pathology. Changes in cardiac metabolism are reflected in the circulation and can be quantified by analysis of blood products. Such metabolic analyses have been performed in studies in ischemic heart disease [14], atherosclerosis [15,16] and heart failure [17], and showed that metabolomic profiling of patient’s blood samples has significant diagnostic and prognostic value [18]. A metabolomic profiling study in patients with severe AS undergoing transcatheter aortic valve replacement (TAVR) showed that circulating long-chain acylcarnitines (LCAC) are associated with the extent of LVH and systolic dysfunction. Furthermore, LCAC levels were reduced within 24 h after TAVR, indicating that relief of pressure overload is directly reflected by a change in circulating metabolites [19].

As prevalence of AS increases with age, the disease is a growing public health burden due to the ageing population [1]. Diagnosis and staging of AS is currently done by physical examination and echocardiography, which can be challenging and requires specialized interpretation. AS is underdiagnosed [20], and if treatment by AVR is delayed, this leads to poor recovery of cardiac function, absence of symptomatic improvement and marked increase of mortality [21,22]. Considering the current limitations to define the stage of AS-induced cardiac remodeling, there is need for a novel method to aid in the diagnosis of AS and timing of intervention, which may be found in metabolomics profiling of patients. Here we studied the metabolic profile of blood serum samples of AS patients and healthy controls to define the major metabolic pathways that are altered in AS patients and assessed the effect of AVR on these metabolites 4 months after surgical intervention.

## 2. Results

### 2.1. Clinical Characteristics of AS Patients Compared to Controls

Clinical characteristics of the study subjects are shown in Supplemental Table S1. AS patients were on average 11 years older than controls. LVM measured by CMR was significantly higher in AS patients compared to controls and was significantly reduced after AVR compared to AS before AVR. Diastolic parameters A, E/A, e’ and E/e’ are elevated in AS patients compared to controls and show a trend towards normalization after AVR. NT-pro-BNP levels are significantly higher in AS patients compared to controls and show a reduction that trends towards significance because of high variation in NT-pro-BNP level among the AS patients. Levels of hemoglobin, kreatinine, ureum, glucose, free fatty acids, and lactate are similar in AS patients before and after AVR.

### 2.2. Metabolic Panel of Thirty Serum Metabolites Distinguishes Severe AS Patients from Healthy Controls

**Figure 1:**
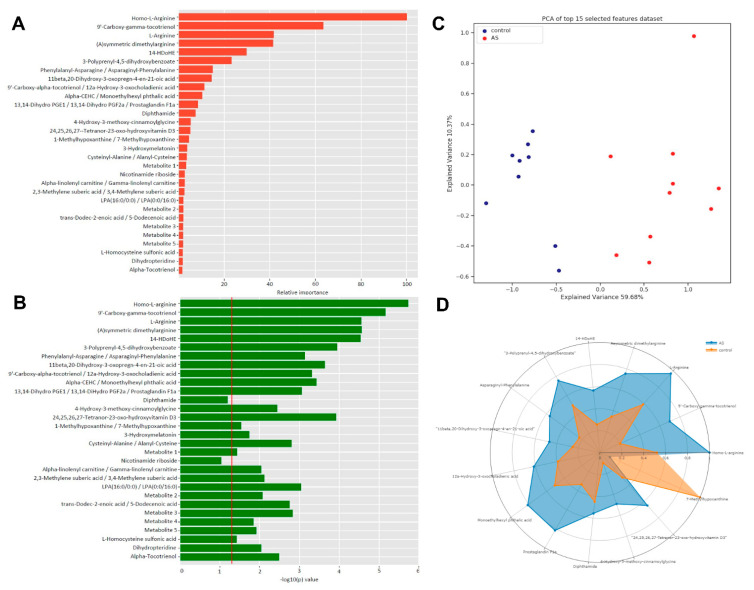
**(A**): The *x*-axis depicts the relative variable importance (%) of a specific metabolite to the distinguishing capacity of the 30 biomarker panel. (**B**): The *x*-axis depicts FDR corrected −10log(p) values, which represents the individual capacity of a metabolite to distinguish between control and AS. (**C**): PLS-DA plot shows good separation between control and AS groups. (**D**): Radar plot of top 15 metabolites visualizes the distinguishing capacity of individual metabolites.

Direct-infusion high-resolution mass spectrometry (DI-HRMS) of control and AS before AVR sera revealed 3851 metabolites corresponding to 1864 unique masses. Multivariate modeling was performed to determine if a metabolic panel could be generated which distinguishes severe AS patients from healthy controls based on their serum metabolome. The model shows good separation by principal component analysis (supplemental Figure S1) and partial least squares-discriminant analysis (Figure 1A). Thirty metabolites were identified which most strongly distinguish severe AS patients from the control group as shown in Figure 1B,C, ranked by their relative importance and with their corresponding −log10(p) values, respectively. Certain metabolites have several isoforms or share the exact mass with other metabolites and could not be uniquely identified by direct-infusion mass spectrometry. The list of possible annotations for these masses is shown in Supplemental Table S2. The radar plot in Figure 1D shows fifteen metabolites of the top 30 metabolite profile and illustrates how these metabolites distinguish AS patients from controls. Overall, the metabolic blood profile is significantly different in AS patients compared to healthy controls.

### 2.3. The Majority of the Top 30 Metabolite Profile Is Associated with Nitric Oxide Metabolism

**Figure 2:**
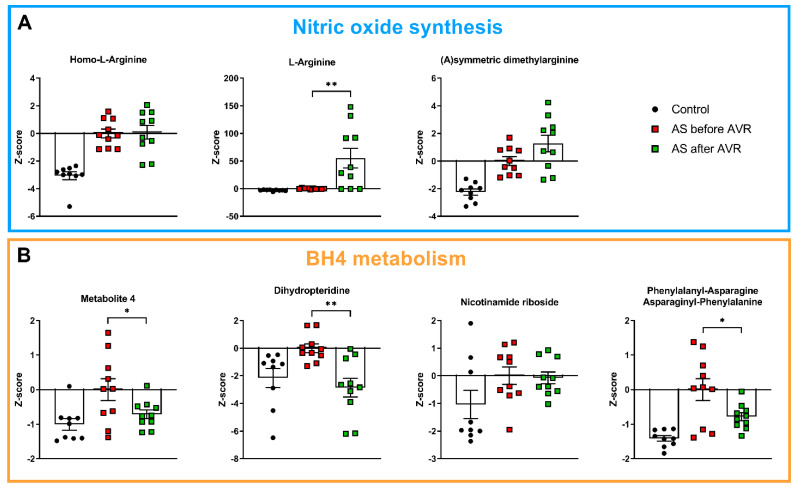
(**A**): Metabolites related to nitric oxide synthesis are higher in AS before AVR compared to controls and show no reversal towards control values after AVR. (**B**): Metabolites related to tetrahydrobiopterin (BH4) metabolism are higher in AS before AVR compared to controls. Metabolite 4, phenylalanyl-asparagine and dihydropteridine show reversal towards control values after AVR. Data shown as mean + standard deviation. * *p* < 0.05, ** *p* < 0.005.

**Figure 3:**
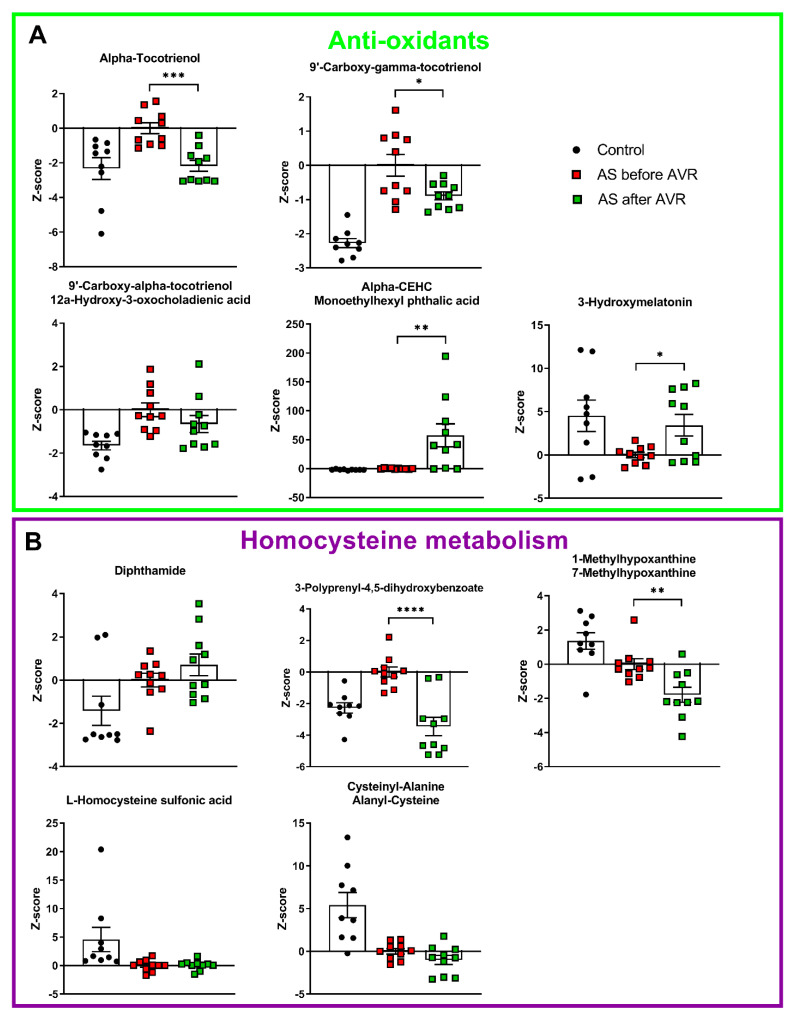
(**A**): 4 intermediates of Vitamine E metabolism, a known anti-oxidant, are increased in AS before AVR compared to controls. 3-hydroxymelatonin is decreased in AS compared to controls. Three tocotrienols and 3-hydroxymelatonin show reversal towards control values after AVR. (**B**): Two metabolites closely related to homocysteine are decreased in AS compared to controls. Of the homocysteine metabolism only 3-polyprenyl-4,5-dihydroxybenzoate shows reversal towards control values after AVR. Data shown as mean + standard deviation. * *p* < 0.05, ** *p* < 0.005, *** *p* < 0.0005, **** *p* < 0.0001.

**Figure 4:**
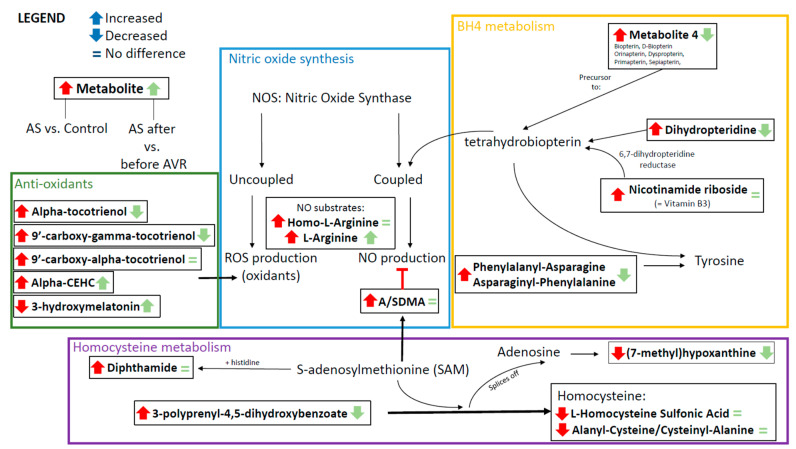
Schematic overview of the connections between metabolites of the metabolic profile, showing interrelationships and changes in AS relative to controls (depicted by the red arrows) and AS before and after AVR (depicted by the green arrows).

Among the thirty main metabolites that distinguish AS patients from controls, 17 are related to nitric oxide (NO) metabolism, and represent NO substrates (Figure 2A), precursors of tetrahydrobiopterin (BH4) (Figure 2B), anti-oxidants (Figure 3A) and homocysteine metabolism (Figure 3B). Their interrelationships and changes in AS relative to controls (depicted by the red arrows) are visualized in Figure 4. In physiological situations, NO is produced from arginine by Nitric Oxide Synthase (NOS) only if coupled by BH4, while uncoupled NOS produces reactive oxygen species (ROS) [23]. Metabolites related to NO production, Homo-L-Arginine and L-Arginine, as well as (a)symmetric dimethylarginine (A/SDMA), which inhibits NO production, all show higher serum levels in AS patients compared to controls (Figure 2A). In addition, precursors of BH4, Metabolite 4, dihydropteridine (BH2) and Nicotinamide riboside, show higher serum levels in AS patients compared to controls (Figure 2B). Moreover, a higher level of phenylalanyl-asparagine is present in AS compared to controls. As pphenylalanine is an essential amino acid, not synthesized de novo in humans and other animals, the high serum level may reflect a disease-mediated increase caused by altered BH4 metabolism.

Four intermediates of vitamin E metabolism have higher serum levels in AS patients compared to controls (Figure 3A): alpha-tocotrienol, 9′-carboxy-gamma-tocotrienol, 9′-carboxy-alpha-tocotrienol/12a-hydroxy-3-oxocholadienic acid, and alpha-CEHC/monoethylhexyl phtalic acid. Vitamin E has an anti-oxidant function. Its main property is the prevention of oxidation of membrane lipids, which stabilizes cell membranes [24]. 3-hydroxymelatonin, also an anti-oxidant, has reduced levels in serum of AS patients compared to controls. These anti-oxidants counteract ROS formation by uncoupled NOS. And finally, five metabolites that are related to homocysteine metabolism, can be linked to NO synthesis. A/SDMA, which inhibits NO production, is formed by the methylation of arginine with s-adenosylmethionine (SAM) as methyl donor and s-adenosylhomocysteine as the demethylated product [25]. Five metabolites that are intimately related to homocysteine metabolism show altered levels in AS patients compared to controls (Figure 3B). SAM and histidine form diphthamide, which has higher serum levels in patients compared to controls. 3-polyprenyl-4,5-dihydroxybenzoate interacts with SAM to form homocysteine by splicing off adenosine which can be further metabolized into (7-methyl)hypoxanthine (Figure 4). Both 3-polyprenyl-4,5-dihydroxybenzoate and (7-methyl)hypoxanthine have higher serum levels in patients compared to controls. Homocysteine-related metabolites L-Homocysteine Sulfonic Acid and Cysteinyl-Alanine/Alanyl-Cysteine show reduced levels in AS patients compared to controls.

### 2.4. AS Patients Show Increased Serum Levels of Fatty Acids and Eicosanoids Indicative for Increased Inflammatory State

**Figure 5:**
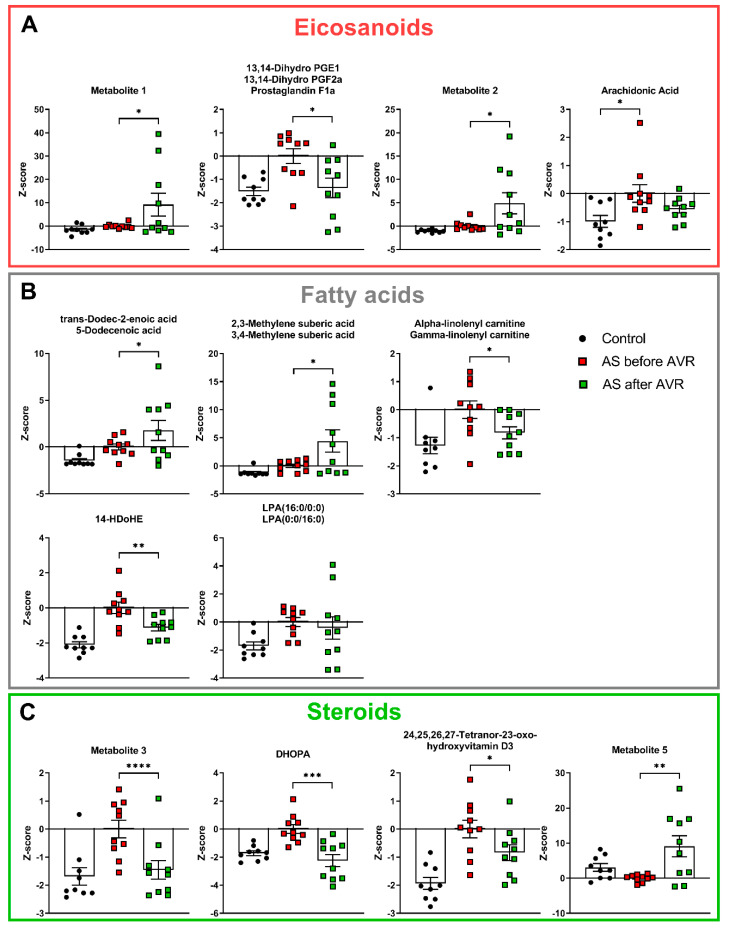
(**A**): All eicosanoids are increased in AS compared to controls. Three eicosanoids show reversal towards control values after AVR. Though Arachidonic acid is not part of the metabolic profile, it shows increased levels in AS compared to controls. (**B**): All fatty acids are increased in AS compared to controls. Three fatty acids show reversal towards control values after AVR. (**C**): All but one steroid is increased in AS compared to controls. All steroids show show reversal towards control values after AVR. DHOPA = 11beta,20-Dihydroxy-3-oxopregn-4-en-21-oic acid. Data shown as mean + standard deviation. * *p* < 0.05, ** *p* < 0.005, *** *p* < 0.0005, **** *p* < 0.0001.

Eight of the top 30 metabolites are related to fatty acids and eicosanoids (Figure 5). All eight metabolites have higher serum levels in AS patients compared to controls. Their relationship to each other is illustrated in Supplemental Figure S2 in a simplified manner for overview purposes. Eicosanoids are synthesized from membrane phospholipids or diacylglycerol (DAG) and play an important role in inflammation [26]. As illustrated in Figure S2, the three eicosanoid metabolites share the exact same mass with many other identified metabolites and are intermediate metabolites of prostaglandins and leukotrienes that are produced from arachidonic acid (AA). Though not present in the top 30 metabolite profile, AA was significantly higher in AS compared to controls (Figure 5A). AA is produced from phospholipids and DAG (Figure S2). Trans-Dodec-2-enoic acid and 3,4-Methylene suberic acid are medium chain fatty acids. Alpha or gamma-linolenyl carnitine is an acylcarnitine, which plays a role in the transport of fatty acids across the mitochondrial membrane for beta-oxidation. 14-HDoHE is an oxidation product of docosahexaenoic acid (DHA), an omega-3 fatty acid.

Lysophosphatidic Acid (LPA (16:0/0:0)) (Figure 5B) is a fatty acid produced by the enzyme phospholipase A2 (PLA2), which also produces eicosanoids from membrane phospholipids. LPA is involved in neutrophil chemotaxis and is a pluripotent lipid mediator of growth, motility, and differentiation released by platelets to influence target cells [27]. Overall, the changes in eicosanoid-related (Figure 5A) and fatty acid-related (Figure 5B) metabolites are indicative for an increased inflammatory state in AS patients.

In line with an altered inflammatory state, four of the top 30 metabolites are steroids and steroid derivatives (Figure 5C), which are known to have inflammation-mediating properties. Three steroids, metabolite 3, 11beta,20-Dihydroxy-3-oxopregn-4-en-21-oic acid (DHOPA) and 24,25,26,27-Tetranor-23-oxo-hydroxyvitamin D3 show increased levels in AS patients compared to controls. Metabolite 3 is a pregnenolone, the first step of the biosynthesis of steroid hormones from cholesterol. DHOPA is a major metabolite of the production of corticosterone. 24,25,26,27-Tetranor-23-oxo-hydroxyvitamin D3 is an intermediate of Vitamin D3 metabolism. Metabolite 5 is a bile acid and shows reduced levels in AS patients. Finally, 4-Hydroxy-3-methoxy-cinnamoylglycine is increased in AS patients compared to controls (Figure S3), but its biological relevance is unknown at this time.

### 2.5. Comparison of Metabolic Panel before and after AVR in Severe AS Patients

To establish if the top 30 metabolites that distinguish AS patients from controls are corrected upon cardiac surgery, metabolomic blood profiling was repeated in the serum of AS patients before and four months after AVR. Supplemental Table S1 illustrates that the heart of AS patients shows reversed remodeling four months after AVR evident from a significant decrease of LV mass and a trend towards normalization of diastolic parameters. Supplemental Table S3 summarizes the changes in the 30 metabolites before (relative to controls) and after AVR (relative to AS before AVR). Paired analyses of metabolites before and after AVR showed a significant change for 21 out of the 30 metabolites, of which 14 changed back towards values observed in controls. Five metabolites show an increase after AVR after an initial increase compared to controls (Figure 2, Figure 3 and Figure 5; Supplemental Table S3).

Before AVR, 17 metabolites associated with NO metabolism were differentially altered in AS compared to controls (13 increased, 4 decreased). Of these 17 metabolites, seven metabolites showed a significant reversal to levels observed in healthy controls 4 months after surgery (Figure 2 and Figure 3; Supplemental Table S3). Compared to the initial change compared to controls, three metabolites involved in BH4 metabolism (Figure 2B), and three of the five anti-oxidants changed in the opposite direction 4 months after AVR (i.e., towards controls), while alpha-CHEC further increased (Figure 3A). Interestingly, among the metabolites associated with NO synthesis and homocysteine metabolism (Figure 2A and Figure 3B, respectively), only one (3-polyprenyl-4,5-dihydroxybenzoate; Figure 3B) showed an opposite change as compared to AS before AVR, while three metabolites significantly increased in the same direction as observed before surgery (Figure 4). The metabolite changes related to NO metabolism four months after AVR (relative to AS before AVR) are depicted with green symbols in Figure 4.

Before AVR, 11 out of the 12 inflammation-related metabolites showed an increased level compared to controls (eicosanoids, fatty acids and steroids; Supplemental Table S3), of which six showed a reduction four months after AVR. Notably, all steroids changed in the direction opposite to the change before surgery in AS patients compared to controls (Figure 5C), indicating normalization of the steroid blood profile four months after surgery.

### 2.6. Higher Endothelial Activation with AS Serum Taken before Relative to after Cardiac Surgery

**Figure 6:**
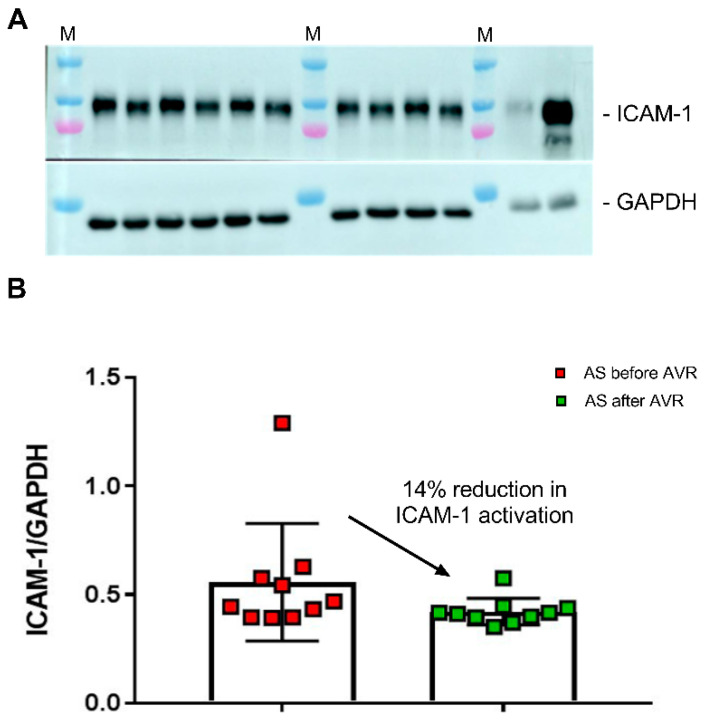
(**A**) Human cardiac microvascular endothelial cells (CMECs) were incubated with all sera from AS patients before (samples indicated with b) and after (samples indicated with a) AVR. As controls, HUVEC cells were incubated without (H0) and with (H6) 10 ng/mL TNF-alpha for 6 h. M, protein marker loaded on the gel. ICAM-1 levels were normalized to GAPDH. (**B**) Values are the mean of two independent incubations for all AS samples taken before and after AVR. A paired one-way t-test showed a significantly lower ICAM-1 level in CMEC exposed to AS after AVR sera compared to AS sera taken before AVR.

Our metabolomics analysis shows that serum from AS patients before AVR is characterized by disturbed NO signaling and increased inflammation, a blood signature which was partially reversed after AVR. To provide evidence that this blood profile may exert a biological effect, we defined the effect of AS patients sera on ICAM-1, a marker of endothelial cell activation in response to increased inflammation/reduced NO signaling in endothelial cells [28]. Thereto, CMECs were incubated for 6 h with sera from AS patients taken before and after AVR (Figure 6A). Our Western blot analyses shows a significant reduction (14%) in ICAM-1 in AS samples taken after AVR compared to AS sera before AVR (Figure 6B).

### 2.7. Correlation between Blood Metabolites and In Vivo Cardiac Efficiency

**Figure 7:**
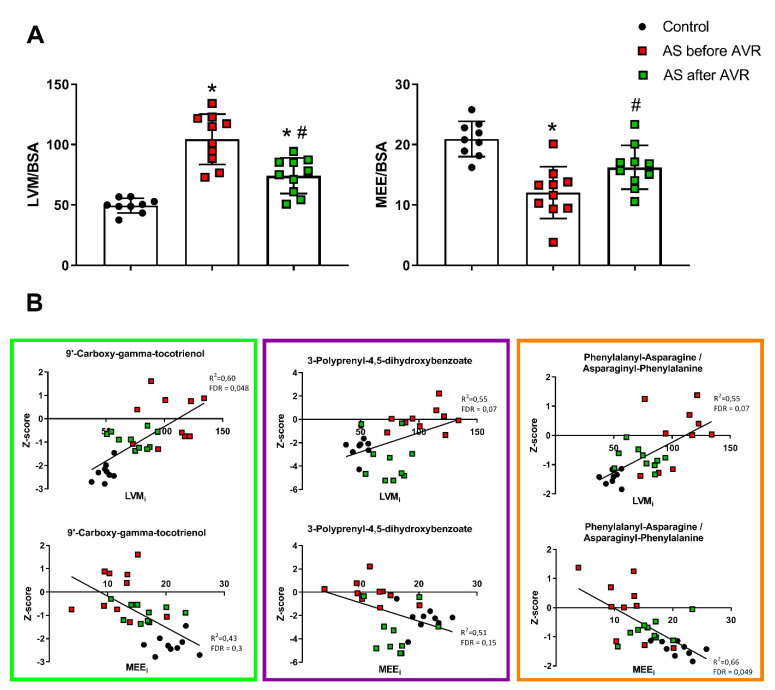
(**A**) Left ventricular mass (LVM) indexed for body surface area (BSA) and myocardial external efficiency (MEE) indexed for BSA. Data shown as mean + standard deviation. * *p* < 0.05 vs. Controls ^#^ *p* < 0.05 vs. AS before AVR. (**B**) Scatterplots of correlations between LVMi and MEEi and 4 metabolites of the top 30 metabolic profile that correlate strongly of which 3 show a pattern of normalization towards control after AVR: 9′-carboxy-gamma-tocotrienol (**left**), 3-polyprenyl-4,5-dihydroxybenzoate (**middle**) and phenylalanyl-asparagine/asparaginyl-phenylalanine (**right**).

To define if the identified metabolic blood profile reflects in vivo cardiac remodeling and efficiency, we correlated the top 30 metabolites with LVM and myocardial external efficiency (MEE) indexed for body surface area (BSA; LVM_i_ and MEE_i_). Our previous study showed hypertrophic remodeling and increased MEE were largely corrected four months after surgery as illustrated in Figure 7A [10]. We performed linear regression analyses between LVM_i_ and MEE_i_ of the control and AS before surgery groups and the top 30 metabolites. After correction for FDR, 9 of the 30 metabolites significantly (using *p* < 0.1 as cutoff value) correlated with LVM_i_ and 1 with MEE_i_ (Supplemental Table S4). Of the nine metabolites that correlate with LVM_i_, three metabolites have an R^2^ > 0.50 (bold text in Supplemental Table S4; Figure 7B) and show a reduction after AVR after an initial increase compared to controls, suggesting that these metabolites reflect reversal of structural (hypertrophy) and functional (efficiency) remodeling. Of these three metabolites, Phenylalanyl-Asparagine/Asparaginyl-Phenylalanine correlates strongly with both LVMi (R^2^ = 0.55, *p* = 0.07) and MEEi (R^2^ = 0.66, *p* = 0.05), 9′-carboxy-gamma-tocotrienol correlates stronger with LVMi (R^2^ = 0.60, *p* = 0.05) than MEEi (R^2^ = 0.43, *p* = 0.3), and also 3-polyprenyl-4,5-dihydroxybenzoate correlates stronger with LVMi (R^2^ = 0.55, *p* = 0.07) than MEEi (R^2^ = 0.51, *p* = 0.16) (Supplemental Table S4; Figure 7B).

### 2.8. Gene Expression Profiles of Myocardial Biopsy of Severe AS Patients Undergoing AVR Show Alterations of Gene Expression of Genes Related to Nitric Oxide Metabolism

To provide additional insight into the potential gene-metabolites interactions in our venous samples, the top 30 metabolic profile was uploaded to Metabridge [29] and mapped to KEGG database for their possible interactive genes. The mRNA expression of each annotated gene per metabolite was checked in a published transcriptome profile comparing cardiac tissues from 18 AS patients with four healthy controls [30]. As can be seen in Supplemental Table S5, expression for genes encoding for Nitric Oxide Synthase is altered in myocardium of AS patients when compared to healthy controls. *NOS1* is upregulated (*p* < 0.05, Log2FoldChange = 0.71) and *NOS3* is downregulated (*p* < 0.005, Log2FoldChange −0.8) in AS compared to healthy controls. The gene *AKR1B10* encoding for aldehyde reductase, which plays a role in BH4 metabolism (Figure 5), is upregulated (*p* < 0.05, Log2FoldChange 0.89) in AS compared to healthy controls. These findings indicate that nitric oxide metabolism is altered on a gene expression level, especially endothelial *NOS3* which is reflected by the increased NO substrates in the serum of AS patients (Figure 2). Multiple genes encoding for various enzymes related to inflammatory molecules of the top 30 metabolic profile show significantly altered expression levels, as can be seen in Supplemental Table S5.

## 3. Discussion

This study performed DI-HRMS-based serum metabolomics and multivariate modeling to determine a metabolic profile of thirty metabolites which distinguishes severe AS patients from healthy controls. Twelve out of thirty blood metabolites, being five anti-oxidants, three intermediates of NO production and four molecules related to BH4 metabolism, associate with the pathomechanism that has been previously described for the AS-mediated cardiac remodeling [2]. This indicates that our metabolic blood profile reflects changes in the heart of AS patients and may serve as biomarkers for disease severity. Although differences exist, AS develops through a process similar to atherosclerosis [2], in which NO, BH4 and oxidative stress play an important role [23,31]. In mice deficient of endothelial nitric oxide synthase (eNOS), atherosclerosis develops at a faster rate [32,33] and upregulation of BH4 synthesis leads to a reduction of atherosclerotic lesions in eNOS overexpressed mice [34]. These findings indicate that NO production by eNOS plays a protective role and uncoupling of eNOS due to a deficiency of BH4 leads to an increase of atherosclerosis. In our metabolic panel, we find that two substrates for NO synthesis and (a)symmetric dimethylarginine, known to inhibit NO synthesis, are increased in AS patients. Furthermore, three metabolites needed for the production of BH4 are increased. Although hypothetical, these findings suggest that in AS the synthesis of NO is disturbed by uncoupled eNOS through a lack of BH4. This in turn leads to the production of ROS, and the need for anti-oxidants, of which five are present in the top 30 metabolic profile. Additionally, gene expression profiles of myocardial biopsy samples of AS patients show that genes encoding for enzymes related to nitric oxide and BH4 metabolism, are significantly altered in AS patients compared to healthy controls. Most notably, the downregulation of *NOS3* (Supplemental Table S5), which is the endothelial type of NOS that plays a specific role in vasodilation.

The second main pathomechanism which is evident from the top metabolic blood profile that distinguishes AS from healthy controls involves the inflammatory response visible from changes in fatty acids, eicosanoids and steroids, which are all elevated in AS patients compared to controls. Additionally, the results of our culture experiments with CMECs showed that the blood of AS patients itself may exert an inflammatory effect. CMECs incubated in AS patient sera before AVR had a significantly higher ICAM-1 activation, a marker for endothelial activation. These findings are in line with studies that showed increased inflammation in AS patients through increased C-reactive protein (CRP) levels [35] and increased macrophage activity measured with 18F-fluordeoxyglucose positron emission tomography which correlates with disease severity [36]. However, CRP does not predict disease progression of calcific aortic valve disease [37], although studies suggest that CRP gene variants may attenuate AS disease progression [38,39]. In line with our study, a metabolomics study in plasma of AS patients found alterations in proteins and metabolites related to inflammation, namely complement and coagulation factors [40].

Components of the metabolic profile correlate strongly with the clinical parameters LVMi and MEEi, indicative for the clinical relevance of the metabolic profile. Three metabolites reflect reversal of structural (hypertrophy) and functional (efficiency) remodeling, namely 9′-carboxy-alpha-tocotrienol, phenylalanyl-asparagine and 3-polyprenyl-4,5-dihydroxybenzoate, the latter is involved in homocysteine metabolism. While homocysteine metabolism is altered in the blood profile of AS patients, apart from 3-polyprenyl-4,5-dihydroxybenzoate, no reversal or even a further deviation from normal was seen after AVR. This in part is in accordance with studies in heart failure and AS where, after initial high expectations [41,42,43], homocysteine levels did not show any predictive value [44]. However, the biological and clinical relation between 3-polyprenyl-4,5-dihydroxybenzoate and AS is strong and requires further research.

Currently, the only available treatment for AS is surgical AVR or TAVR, while there is no effective preventative therapy. Research is being done into the pathomechanism of CAVD and how to intervene pharmacologically in order to halt CAVD progression, preferably in an early disease stage. An animal study showed that rabbits on high cholesterol diet treated with atorvastatin showed decreased aortic valve calcification and increased eNOS production compared to controls [45], which is of particular interest considering eNOS seems to play a key role in AS based on the top 30 metabolic profile identified by our study. Statins have been suggested to slow AS progression in observational studies [46,47,48], but randomized clinical trials showed no difference between statin treatment and placebo in AS reduction [49,50,51,52]. There is considerable evidence that lipid metabolism plays an incremental role in the development and progression of CAVD. Phospholipids (PL), low density lipoprotein (LDL), and lysophosphatidic acids and their oxidized forms have been associated with CAVD and AS in numerous studies [53,54,55,56,57], and we have found five anti-oxidants and eight metabolites in our metabolic profile that can be directly linked to lipid metabolism. We expect to find more of these associations in the future, as DI-HRMS is limited in its capacity to detect lipids due to their size and the used extraction methods. In the future we will perform lipidomics to further elucidate the role of lipids in blood biomarker-based assessment of CAVD and AS. A limitation to our current study is that comparisons between AS and control group were not corrected for age.

Our study defined a top 30 metabolic profile that reflects biologically relevant mechanisms of CAVD and AS and correlated with LV mass and cardiac efficiency. Future studies should include aortic root and coronary sinus sampling to more accurately analyze blood metabolites directly before and after perfusion of the heart. Gene expression profiles of the aortic valve could also provide more information on the origin of the metabolites found in our metabolic profile. Longitudinal studies in AS patients with different disease severities (mild, moderate, severe stenosis) are warranted to build a risk prediction model that is based on the top 30 metabolites profile and identify AS patients in need of cardiac surgery.

## 4. Methods

### 4.1. Study Population

**Figure 8:**
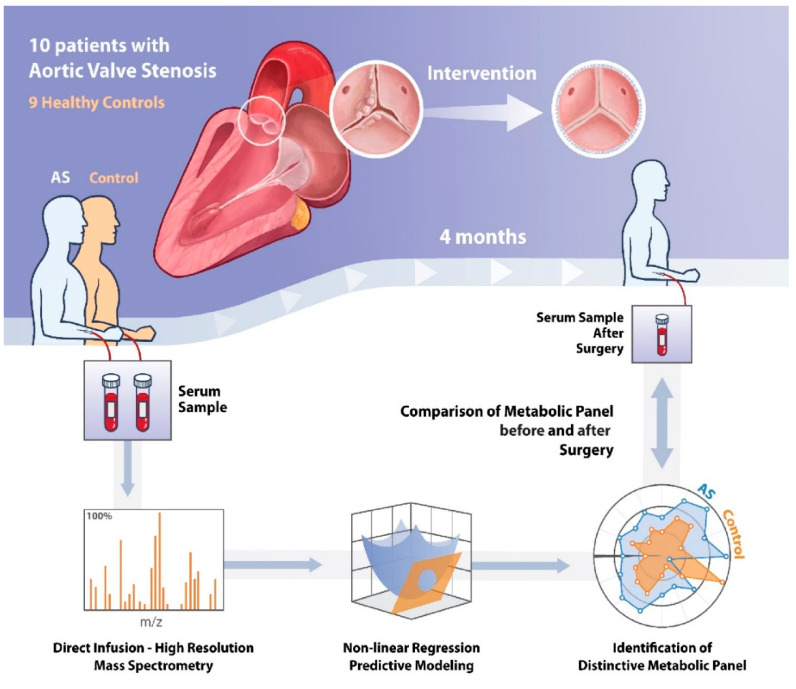
Schematic illustration of the methods of this study. AS = Aortic Stenosis.

The study population consisted of 19 subjects: 10 patients with AS and 9 healthy controls (Figure 8). Fasted venous samples were collected from controls, and from AS patients before and 4 months after surgical intervention. Study exclusion criteria were previously documented and were as follows: aortic regurgitation grade >1, coronary artery disease (coronary artery stenosis >30%), previous cardiac surgery, ejection fraction <50%, diabetes mellitus or hypertension, and estimated glomerular filtration rate <30 mL/min per 1.73∙m^2^ [9].

### 4.2. Blood Serum Preparation

Individuals fasted overnight prior to collection of blood samples. The samples were collected by venous puncture and allowed to clot for 1 h at 37 °C. Following this incubation period, clotted samples were left to contract for at least 30 min at 4 °C. The serum was centrifuged (4000 rpm, 20 min, 4 °C) and separated. Sodium azide (0.01%) was added to the supernatant. All samples were stored at −20 °C until use.

### 4.3. Metabolomics Profiling

Analysis of the metabolites was conducted as previously described [58]. Briefly, a non-quantitative direct-infusion high-resolution mass spectrometry (DI-HRMS) based metabolomics method was applied in combination with a nano-electrospray ionization source. The mean peak intensities of the technical triplicates were calculated and annotated by matching the m/z of these mass peaks with a range of two parts per million to metabolite masses that are found in the Human Metabolome Database [59]. Accordingly, mass peaks were identified. Serum samples from controls and AS, and AS before and after AVR were run in two separate DI-HRMS runs. To allow comparison between the two runs, individual metabolite values were transformed into Z-scores, with the units being the standard deviation away from the mean of AS before AVR as these samples were included in both runs.

### 4.4. Culture Experiments

Human cardiac microvascular endothelial cells (CMECs, Lonza, CC-7030) were cultured and tested negative for mycoplasma as detected using the MycoAlert assay (Mycoalert Mycoplasma Detection Kit, Lonza, LT07-418; MycoAlert assay control set, Lonza, LT07-518). The cells were grown in endothelial growth medium (EGM)^TM^-2MV (CC-3202, Lonza), containing endothelial cell basal medium (CC-3156, Lonza) supplemented with EGM^TM^-2MV microvascular endothelial cell growth medium singlequots^TM^ kit (CC-4147, Lonza). Confluent CMECs were incubated for 6 h at 37 °C and 5% CO_2_ with EGM^TM^-2MV (without FBS) supplemented with 15% AS serum. As positive control, HUVEC (human umbilical vein endothelial cells) were incubated for 6 h in medium which was supplemented with the inflammatory mediator TNF-α (10 ng/mL).

CMEC-incubations with patient serum were performed in duplo. After incubation, the medium was removed and endothelial cells were washed 1 time with PBS. Subsequently, ECs were put in 2× sample buffer and samples were loaded on an 8% gel and transferred to a blot and stained overnight with specific antibodies against ICAM-1 (Santa Cruz, sc8439, dilution 1:1000) and GAPHDH (Cell Signaling, 2118, 1:10,000). Secondary IgG-horseradish peroxidase (HRP)-conjugated antibodies were applied for 2 h at room temperature. Images were generated using ECL Prime (Amersham, RPN2232) and the LAS-3000 documentation system (Fuji Film Life Science, Cambridge, MA, USA).

### 4.5. Data Analysis and Modeling

We identified panels of metabolic biomarkers in order to discriminate between the AS and control group. In brief, we used a combination of multiple gradient boosting classifiers to improve prediction accuracy [60]. To avoid over-fitting, we used a 5-fold stratified cross-validation over the training partition of the data (80%) while the remaining data (20%) was used as the test dataset. We conducted a rigorous stability selection procedure to ensure the reliability and robustness of the biomarker signatures [61]. This was repeated 10 times and Receiver-Operating-Characteristics Area-Under-Curve (ROC AUC) scores were computed each time and averaged for the final test ROC AUC. A permutation (randomization test) was used to evaluate statistical validity of the results [62]. In the permutation test, the outcome variable was randomly reshuffled 1000 times while the corresponding -omics profiles were kept intact. We used Python v. 3.8 (www.python.org), with packages Numpy, Scipy and Scikits-learn for implementing the model and R version 3.5.3 for visualizations.

Differences in serum levels of the metabolic panel metabolites before and after AVR were assessed by one-tailed paired t-tests. Linear regression analysis was performed using Spearman correlation and *p*-values were corrected for false discovery rate (FDR) using the Benjami-Hochberg correction. A *p*-value < 0.1 for FDR corrected *p*-values is commonly considered as significantly different.

### 4.6. Cardiovascular Magnetic Resonance (CMR) and [^11^C]-Acetate Positron Emission Tomography (PET)

CMR was performed on a 1.5 T whole body scanner (Magnetom Sonata or Avanto, Siemens, Erlangen, Germany), using a six-channel phased-array body coil. A contiguous, short-axis, steady-state free precession stack was acquired extending from the mitral valve annulus to the LV apex, to obtain global LV function parameters, including end-diastolic volume (LVEDV), end-systolic volume (LVESV), stroke volume (SV), LV ejection fraction (LVEF), and LV mass (LVM).

Myocardial energy efficiency (MEE) was calculated using a combination of parameters obtained by CMR and [^11^C]-acetate scans obtained on a Gemini TF-64 PET/CT scanner (Philips Healthcare, Best, The Netherlands). A detailed version of the methods describing the protocol and accuracy regarding the calculation of MEE can be found in the online supplement of earlier research by our group [10].

## Data Availability

The data presented in this study are available on request from the corresponding author. The data are not publicly available as consent for publishing of personal data was not asked at the time.

## Supplementary Materials

The following are available online at https://www.mdpi.com/article/10.3390/ijms22073569/s1, Figure S1: Principal component analysis (PCA) plot shows good separation of the model between controls and AS patients, Figure S2, Figure S3: 4-hydroxy-3-methoxy-cinnamoylglycine is of unknownbiologicalrelevance. Table S1: characteristics of all study subjects, Table S2: List of possible annotations for identical masses, Table S3: Overview of top 30 metabolite changes in AS patients relative to controls (before AVR)(shown in Figure 2), and changes 4 months after AVR relative to AS before AVR, Table S4: Correlation between the top 30 metabolites of the metabolic profile and left ventricular mass (LVM) indexed for body surface area (BSA) and myocardial external efficiency indexed for BSA, respectively LVMi and MEEi.

## References

[1] Yadgir S., Johnson C.O., Aboyans V., Adebayo O.M., Adedoyin R.A., Afarideh M., Alahdab F., Alashi A., Alipour V., Arabloo J. (2020). Global, Regional, and National Burden of Calcific Aortic Valve and Degenerative Mitral Valve Diseases, 1990–2017. Circulation.

[2] Dweck M.R., Boon N.A., Newby D.E. (2012). Calcific Aortic Stenosis: A Disease of the Valve and the Myocardium. J. Am. Coll. Cardiol..

[3] Leon M.B., Smith C.R., Mack M.J., Miller D.C., Moses J.W., Svensson L.G., Tuzcu E.M., Webb J.G., Fontana G.P., Makkar R. (2010). Transcatheter Aortic-Valve Implantation for Aortic Stenosis in Patients Who Cannot Undergo Surgery. N. Engl. J. Med..

[4] Hein S., Arnon E., Kostin S., Schönburg M., Elsässer A., Polyakova V., Bauer E.P., Klövekorn W.P., Schaper J. (2003). Progression from compensated hypertrophy to failure in the pressure-overloaded human: Heart structural deterioration and compensatory mechanisms. Circulation.

[5] Ingwall J.S. (2006). On the hypothesis that the failing heart is energy starved: Lessons learned from the metabolism of ATP and creatine. Curr. Hypertens. Rep..

[6] Ingwall J.S., Atkinson D.E., Clarke K., Fetters J.K. (1990). Energetic correlates of cardiac failure: Changes in the creatine kinase system in the failing myocardium. Eur. Hear. J..

[7] Neubauer S., Horn M., Cramer M., Harre K., Newell J.B., Peters W., Pabst T., Ertl G., Hahn D., Ingwall J.S. (1997). Myocardial Phosphocreatine-to-ATP Ratio Is a Predictor of Mortality in Patients with Dilated Cardiomyopathy. Circulation.

[8] Weiss R.G., Gerstenblith G., Bottomley P.A. (2005). ATP flux through creatine kinase in the normal, stressed, and failing human heart. Proc. Natl. Acad. Sci. USA.

[9] Güçlü A., Knaapen P., Harms H.J., Parbhudayal R.Y., Michels M., Lammertsma A.A., Van Rossum A.C., Germans T., Van Der Velden J. (2017). Disease Stage–Dependent Changes in Cardiac Contractile Performance and Oxygen Utilization Underlie Reduced Myocardial Efficiency in Human Inherited Hypertrophic Cardiomyopathy. Circ. Cardiovasc. Imaging.

[10] Güçlü A., Knaapen P., Harms H.J., Vonk A.B., Stooker W., Groepenhoff H., Lammertsma A.A., Van Rossum A.C., Germans T., Van Der Velden J. (2015). Myocardial efficiency is an important determinant of functional improvement after aortic valve replacement in aortic valve stenosis patients: A combined PET and CMR study. Eur. Hear. J. Cardiovasc. Imaging.

[11] Korvald C., Elvenes O.P., Myrmel T. (2000). Myocardial substrate metabolism influences left ventricular energetics in vivo. Am. J. Physiol. Circ. Physiol..

[12] Zhang L., Jaswal J.S., Ussher J.R., Sankaralingam S., Wagg C., Zaugg M., Lopaschuk G.D. (2013). Cardiac Insulin-Resistance and Decreased Mitochondrial Energy Production Precede the Development of Systolic Heart Failure After Pressure-Overload Hypertrophy. Circ. Hear. Fail..

[13] Taegtmeyer H., Young M.E., Lopaschuk G.D., Abel E.D., Brunengraber H., Darley-Usmar V., Rosiers C.D., Gerszten R., Glatz J.F., Griffin J.L. (2016). Assessing Cardiac Metabolism: A Scientific Statement from the American Heart Association. Circ. Res..

[14] Sabatine M.S., Liu E., Morrow D.A., Heller E., McCarroll R., Wiegand R., Berriz G.F., Roth F.P., Gerszten R.E. (2005). Metabolomic Identification of Novel Biomarkers of Myocardial Ischemia. Circulation.

[15] Zhu W., Gregory J.C., Org E., Buffa J.A., Gupta N., Wang Z., Li L., Fu X., Wu Y., Mehrabian M. (2016). Gut Microbial Metabolite TMAO Enhances Platelet Hyperreactivity and Thrombosis Risk. Cell.

[16] Tang W.W., Hazen S.L. (2014). The contributory role of gut microbiota in cardiovascular disease. J. Clin. Investig..

[17] Ahmad T., Kelly J.P., McGarrah R.W., Hellkamp A.S., Fiuzat M., Testani J.M., Wang T.S., Verma A., Samsky M.D., Donahue M.P. (2016). Prognostic Implications of Long-Chain Acylcarnitines in Heart Failure and Reversibility with Mechanical Circulatory Support. J. Am. Coll. Cardiol..

[18] Nascimben L., Ingwall J.S., Pauletto P., Friedrich J., Gwathmey J.K., Saks V., Pessina A.C., Allen P. (1996). Creatine Kinase System in Failing and Nonfailing Human Myocardium. Circulation.

[19] Elmariah S., Farrell L.A., Furman D., Lindman B.R., Shi X., Morningstar J.E., Rhee E.P., Gerszten R.E. (2018). Association of Acylcarnitines With Left Ventricular Remodeling in Patients with Severe Aortic Stenosis Undergoing Transcatheter Aortic Valve Replacement. JAMA Cardiol..

[20] D’Arcy J.L., Coffey S., Loudon M.A., Kennedy A., Pearson-Stuttard J., Birks J., Frangou E., Farmer A.J., Mant D., Wilson J. (2016). Large-scale community echocardiographic screening reveals a major burden of undiagnosed valvular heart disease in older people: The OxVALVE Population Cohort Study. Eur. Hear. J..

[21] Elmariah S., Palacios I.F., McAndrew T., Hueter I., Inglessis I., Baker J.N. (2013). Outcomes of transcatheter and surgical aortic valve replacement in high-risk patients with aortic stenosis and left ventricular dysfunction: Results from the Placement of Aortic Transcatheter Valves (PARTNER) trial (cohort A). Circ. Cardiovasc. Interv..

[22] Lindman B.R., Stewart W.J., Pibarot P., Hahn R.T., Otto C.M., Xu K., Devereux R.B., Weissman N.J., Enriquez-Sarano M., Szeto W.Y. (2014). Early Regression of Severe Left Ventricular Hypertrophy After Transcatheter Aortic Valve Replacement Is Associated With Decreased Hospitalizations. JACC Cardiovasc. Interv..

[23] Förstermann U., Sessa W.C. (2012). Nitric oxide synthases: Regulation and function. Eur. Heart J..

[24] Zingg J.-M. (2018). Vitamin E: Regulatory Role on Signal Transduction. IUBMB Life.

[25] Van Guldener C., Nanayakkara P.W., Stehouwer C.D. (2007). Homocysteine and asymmetric dimethylarginine (ADMA): Biochemically linked but differently related to vascular disease in chronic kidney disease. Clin. Chem. Lab. Med. (CCLM).

[26] Zeldin D.C. (2001). Epoxygenase Pathways of Arachidonic Acid Metabolism. J. Biol. Chem..

[27] Gerrard J.M., Clawson C.C., White J.G. (1980). Lysophosphatidic acids: III. Enhancement of neutrophil chemotaxis. Am. J. Pathol..

[28] Lawson C., Wolf S. (2009). ICAM-1 signaling in endothelial cells. Pharmacol. Rep..

[29] Hinshaw S.J., Lee A.H.Y., E Gill E., Hancock R.E.W. (2018). MetaBridge: Enabling network-based integrative analysis via direct protein interactors of metabolites. Bioinformatics.

[30] Pei J., Harakalova M., Treibel T.A., Lumbers R.T., Boukens B.J., Efimov I.R., Van Dinter J.T., González A., López B., El Azzouzi H. (2020). H3K27ac acetylome signatures reveal the epigenomic reorganization in remodeled non-failing human hearts. Clin. Epigenet..

[31] Förstermann U., Xia N., Li H. (2017). Roles of Vascular Oxidative Stress and Nitric Oxide in the Pathogenesis of Atherosclerosis. Circ. Res..

[32] Chen J., Kuhlencordt P.J., Astern J., Gyurko R., Huang P.L. (2001). Hypertension Does Not Account for the Accelerated Atherosclerosis and Development of Aneurysms in Male Apolipoprotein E/Endothelial Nitric Oxide Synthase Double Knockout Mice. Circulation.

[33] Kuhlencordt P.J., Gyurko R., Han F., Scherrer-Crosbie M., Aretz T.H., Hajjar R. (2001). Accelerated Atherosclerosis, Aortic Aneurysm Formation, and Ischemic Heart Disease in Apolipoprotein E/Endothelial Nitric Oxide Synthase Double-Knockout Mice. Circulation.

[34] Takaya T., Hirata K.I., Yamashita T., Shinohara M., Sasaki N., Inoue N. (2007). A Specific Role for eNOS-Derived Reactive Oxygen Species in Atherosclerosis Progression. Arterioscler. Thromb. Vasc. Biol..

[35] Galante A., Pietroiusti A., Vellini M., Piccolo P., Possati G., De Bonis M., Grillo R.L., Fontana C., Favalli C. (2001). C-reactive protein is increased in patients with degenerative aortic valvular stenosis. J. Am. Coll. Cardiol..

[36] Dweck M.R., Jones C., Joshi N.V., Fletcher A.M., Richardson H., White A., Marsden M., Pessotto R., Clark J.C., Wallace W.A. (2012). Assessment of Valvular Calcification and Inflammation by Positron Emission Tomography in Patients with Aortic Stenosis. Circulation.

[37] Novaro G.M., Katz R., Aviles R.J., Gottdiener J.S., Cushman M., Psaty B.M. (2007). Clinical factors, but not C-reactive protein, predict progression of calcific aortic-valve disease: The Cardiovascular Health Study. J. Am. Coll. Cardiol..

[38] Wypasek E., Potaczek D.P., Lamplmayr M., Sadowski J., Undas A. (2014). Interleukin-6 receptor Asp358Ala gene polymorphism is associated with plasma C-reactive protein levels and severity of aortic valve stenosis. Clin. Chem. Lab. Med..

[39] Wypasek E., Potaczek D.P., Undas A. (2015). Association of the C-Reactive Protein Gene (CRP) rs1205 C>T Polymorphism with Aortic Valve Calcification in Patients with Aortic Stenosis. Int. J. Mol. Sci..

[40] Mourino-Alvarez L., Baldan-Martin M., Gonzalez-Calero L., Martinez-Laborde C., Sastre-Oliva T., Moreno-Luna R., Lopez-Almodovar L.F., Sanchez P.L., Fernandez-Aviles F., Vivanco F. (2016). Patients with calcific aortic stenosis exhibit systemic molecular evidence of ischemia, enhanced coagulation, oxidative stress and impaired cholesterol transport. Int. J. Cardiol..

[41] Herrmann M., Taban-Shomal O., Hübner U., Böhm M., Herrmann W. (2006). A review of homocysteine and heart failure. Eur. J. Hear. Fail..

[42] Sundström J., Vasan R.S. (2005). Homocysteine and heart failure: A review of investigations from the Framingham Heart Study. Clin. Chem. Lab. Med..

[43] Novaro G.M., Aronow H.D., Mayer-Sabik E., Griffin B.P. (2004). Plasma homocysteine and calcific aortic valve disease. Heart.

[44] Wu G., Xian J., Yang X., Li J., Liu J., Dong W., Su S., Li J., Tu Y., Peng J. (2018). Association between homocysteine levels and calcific aortic valve disease: A systematic review and meta-analysis. Oncotarget.

[45] Rajamannan N.M., Subramaniam M., Stock S.R., Stone N.J., Springett M., I Ignatiev K., McConnell J.P., Singh R., O Bonow R., Spelsberg T.C. (2005). Atorvastatin inhibits calcification and enhances nitric oxide synthase production in the hypercholesterolaemic aortic valve. Heart.

[46] Novaro G.M., Tiong I.Y., Pearce G.L., Lauer M.S., Sprecher D.L., Griffin B.P. (2001). Effect of Hydroxymethylglutaryl Coenzyme A Reductase Inhibitors on the Progression of Calcific Aortic Stenosis. Circulation.

[47] Rajamannan N.M., Subramaniam M., Caira F., Stock S.R., Spelsberg T.C. (2005). Atorvastatin inhibits hypercholesterolemia-induced calcification in the aortic valves via the Lrp5 receptor pathway. Circulation.

[48] Parolari A., Tremoli E., Cavallotti L., Trezzi M., Kassem S., Loardi C., Veglia F., Ferrari G., Pacini D., Alamanni F. (2011). Do statins improve outcomes and delay the progression of non-rheumatic calcific aortic stenosis?. Heart.

[49] Chan K.L., Teo K., Dumesnil J.G., Ni A., Tam J. (2010). Effect of Lipid lowering with rosuvastatin on progression of aortic stenosis: Results of the aortic stenosis progression observation: Measuring effects of rosuvastatin (ASTRONOMER) trial. Circulation.

[50] Cowell S.J., Newby D.E., Prescott R.J., Bloomfield P., Reid J., Northridge D.B., Boon N.A. (2005). A Randomized Trial of Intensive Lipid-Lowering Therapy in Calcific Aortic Stenosis. N. Engl. J. Med..

[51] Rossebø A.B., Pedersen T.R., Boman K., Brudi P., Chambers J.B., Egstrup K., Gerdts E., Gohlke-Bärwolf C., Holme I., Kesäniemi Y.A. (2008). Intensive Lipid Lowering with Simvastatin and Ezetimibe in Aortic Stenosis. N. Engl. J. Med..

[52] Dichtl W., Alber H.F., Feuchtner G.M., Hintringer F., Reinthaler M., Bartel T., Süssenbacher A., Grander W., Ulmer H., Pachinger O. (2008). Prognosis and Risk Factors in Patients with Asymptomatic Aortic Stenosis and Their Modulation by Atorvastatin (20 mg). Am. J. Cardiol..

[53] Capoulade R., Chan K.L., Yeang C., Mathieu P., Bossé Y., Dumesnil J.G., Tam J.W., Teo K.K., Mahmut A., Yang X. (2015). Oxidized Phospholipids, Lipoprotein(a), and Progression of Calcific Aortic Valve Stenosis. J. Am. Coll. Cardiol..

[54] Olsson M., Thyberg J., Nilsson J. (1999). Presence of Oxidized Low Density Lipoprotein in Nonrheumatic Stenotic Aortic Valves. Arter. Thromb. Vasc. Biol..

[55] Nsaibia M.J., Boulanger M.C., Bouchareb R., Mkannez G., Le Quang K., Hadji F. (2017). OxLDL-derived lysophosphatidic acid promotes the progression of aortic valve stenosis through a LPAR1-RhoA-NF-κB pathway. Cardiovasc. Res..

[56] Cote C., Pibarot P., Despres J.-P., Mohty D., Cartier A., Arsenault B.J., Couture C., Mathieu P. (2008). Association between circulating oxidised low-density lipoprotein and fibrocalcific remodelling of the aortic valve in aortic stenosis. Heart.

[57] Kamstrup P.R., Hung M.Y., Witztum J.L., Tsimikas S., Nordestgaard B.G. (2017). Oxidized Phospholipids and Risk of Calcific Aortic Valve Disease: The Copenhagen General Population Study. Arterioscler. Thromb. Vasc. Biol..

[58] Haijes H.A., Willemsen M., Van Der Ham M., Gerrits J., Pras-Raves M.L., Prinsen H.C.M.T., Van Hasselt P.M., Velden M.G.M.D.S.-V.D., Verhoeven-Duif N.M., Jans J.J.M. (2019). Direct Infusion Based Metabolomics Identifies Metabolic Disease in Patients’ Dried Blood Spots and Plasma. Metabolics.

[59] Wishart D.S., Feunang Y.D., Marcu A., Guo A.C., Liang K., Vázquez-Fresno R., Sajed T., Johnson D., Allison P., Karu N. (2018). HMDB 4.0: The human metabolome database for 2018. Nucleic Acids Res..

[60] Caruana R., Niculescu-Mizil A., Crew G., Ksikes A. (2004). Ensemble selection from libraries of models. Proceedings of the Twenty-First International Conference on Machine Learning—ICML ’04.

[61] Chen T., Guestrin C. XGBoost: A Scalable Tree Boosting System. Proceedings of the 22nd ACM SIGKDD International Conference on Knowledge Discovery and Data Mining.

[62] Meinshausen N., Bühlmann P. (2010). Stability selection. J. R. Stat. Soc. Ser. B Stat. Methodol..

