# Supplementary material for: Metabolomics in Severe Aortic Stenosis Reveals Intermediates of Nitric Oxide Synthesis as Most Distinctive Markers
Source: Int J Mol Sci. 2021 Mar 30;22(7):3569. doi: 10.3390/ijms22073569 (PMC8037707; doi:10.3390/ijms22073569)
Supplement: Supplementary file 1 [file ijms-22-03569-s001.zip › Supplement.pdf]

**Supplemental table 1.** Characteristics of all study subjects.

| | Control (n=9) | AS before AVR (n=10) | AS after AVR (n=10) |
|---|---|---|---|
| **Age (years)** | 51 ± 9 | 62 ± 10* | 62 ± 10 |
| **Men (%)** | 6 (67%) | 7 (70%) | 7 (70%) |
| **BMI (kg/m$^2$)** | 26 ± 3 | 28 ± 3 | 28 ± 4 |
| **Heart rate (beats per minute)** | 66 ± 10 | 66 ± 7 | 66 ± 6 |
| **Systolic blood pressure (mm Hg)** | 124 ± 14 | 120 ± 11 | 122 ± 12 |
| **Diastolic blood pressure (mm Hg)** | 69 ± 4 | 69 ± 10 | 72 ± 9 |
| *Cardiac parameters* | | | |
| **LVEF (%)** | 61 ± 6 | 58 ± 7 | 61 ± 5 |
| **LVEDV (mL)** | 201 ± 42 | 202 ± 46 | 178 ± 40 |
| **LVESV (mL)** | 79 ± 25 | 87 ± 32 | 71 ± 21 |
| **LV mass** | 103 ± 18 | 209 ± 50* | 151 ± 38$^{\#}$ |
| **E** | 78 ± 11 | 72 ± 17 | 81 ± 13 |
| **A** | 60 ± 10 | 76 ± 15* | 77 ± 17 |
| **E/A** | 0.98 ± 0,3 | 1.34 ± 0,3* | 1.1 ± 0,3 |
| **Septal e'** | 9 ± 2,1 | 6 ± 2* | 8 ± 1$^{\#}$ |
| **Lateral e'** | 14 ± 3 | 6 ± 2* | 10 ± 2$^{\#}$ |
| **E/e'** | 7 ± 1 | 12 ± 3* | 10 ± 2$^{\#}$ |
| *Laboratory data* | | | |
| **Hemoglobin (mmol/L)** | 8 ± 0,5 | 9 ± 0,7* | 9 ± 1 |
| **Hematocrit** | 0.39 ± 0,02 | 0.43 ± 0,04* | 0.42 ± 0,05 |
| **Kreatinine (mmol/L)** | 94 ± 8 | 74 ± 10* | 78 ± 13 |
| **Ureum (mmol/L)** | 5 ± 1 | 6 ± 1 | 6 ± 1 |
| **Glucose (mmol/L)** | 6 ± 0,4 | 6 ± 0,7 | 6 ± 0,6 |
| **Free fatty acids (mmol/L)** | 0.5 ± 0,3 | 0.7 ± 0,3 | 0.6 ± 0,2 |
| **Lactate (mmol/L)** | 1 ± 0,6 | 1 ± 0,5 | 2 ± 1 |
| **NT-pro-BNP** | 54 ± 58 | 439 ± 537* | 247 ± 151 |

Values are depicted as mean ± SD.
BMI = Body mass index, LVEF = Left Ventricular Ejection Fraction, LVEDV = Left Ventricular End Diastolic Volume, LVESV = Left Ventricular End Systolic Volume, E = early diastolic mitral flow velocity, A = late diastolic mitral flow velocity, e´ = early diastolic mitral annulus velocity, NT-pro-BNP = N-terminal fragment B-type natriuretic peptide. *=$p<0.05$ AS before AVR compared to control, $^{\#}$=$p<0.05$ AS after AVR compared to AS before AVR.

**Supplemental table 2.** List of possible annotations for identical masses.

| Metabolite 1 | Metabolite 2 | Metabolite 3 | Metabolite 4 | Metabolite 5 |
|---|---|---|---|---|
| 5(S)-Hydroperoxy-eicosatetraenoic acid | 8,9-Epoxyeicosatrienoic acid | 17a-Hydroxypregnenolone | Biopterin | 5b-Cholestane-3a,7a,12a,25-tetrol |
| 8-iso-PGA1 | 14R,15S-EpETrE | 3a,7a-Dihydroxy-5b-cholestanate | D-Biopterin | Cholestane-3,7,12,25-tetrol |
| Prostaglandin A1 | 15(S)-HETE | 21-Hydroxypregnenolone | Orinapterin | 27-Deoxy-5b-cyprinol |
| Prostaglandin B1 | 14,15-Epoxy-5,8,11-eicosatrienoic acid | 17-alpha,20-alpha-Dihydroxypregn-4-en-3-one | Dyspropterin | 5b-Cholestane-3a,7a,12a,23-Tetrol |
| 12(S)-HPETE | 11,12-Epoxyeicosatrienoic acid | 5alpha-Dihydrodeoxycorticosterone | Primapterin | 5a-Cholestane-3a,7a,12a,25-tetrol |
| 15(S)-HPETE | 8-HETE | 7alpha-Hydroxypregnenolone | Sepiapterin | |
| Hepoxilin A3 | 16(R)-HETE | 16-a-Hydroxypregnenolone | | |
| Hepoxilin B3 | 11(R)-HETE | | | |
| 12(R)-HPETE | 20-Hydroxyeicosa-tetraenoic acid | | | |
| 11H-14,15-EETA | 12-HETE | | | |
| 11(R)-HPETE | 18-Hydroxyarachidonic acid | | | |
| 8(S)-HPETE | 9-HETE | | | |
| 15H-11,12-EETA | 11,12-EpETrE | | | |
| 6-trans-Leukotriene B4 | 5-HETE | | | |
| 6-trans-12-epi-Leukotriene B4 | 19(S)-HETE | | | |
| 12(S)-Leukotriene B4 | 10-HETE | | | |
| 14,15-DiHETE | 13-HETE | | | |
| 17,18-DiHETE | 17-HETE | | | |
| 5,15-DiHETE | 12 Hydroxy arachidonic acid | | | |
| 8,15-DiHETE | Arachidonate | | | |
| 5-HPETE | 5,6-Epoxy-8,11,14-eicosatrienoic acid | | | |
| 10,11-dihydro-12-oxo-LTB4 | | | | |
| 6,7-dihydro-5-oxo-12-epi-LTB4 | | | | |
| 9-Deoxy-delta12-PGD2 | | | | |
| Prostaglandin C1 | | | | |
| 12,20-DiHETE | | | | |
| Leukotriene B4 | | | | |

**Supplemental Table 3.** Overview of top 30 metabolite changes in AS patients relative to controls (before AVR)(shown in Figure 2), and changes 4 months after AVR relative to AS before AVR.

| | Metabolite | Before AVR | After AVR | p value Before vs after AVR |
|---|---|---|---|---|
| **Nitric oxide synthesis** | Homo-L-Arginine | ↑ | = | 0.45 |
| | D-Arginine / L-Arginine | ↑ | ↑ | 0.006* |
| | (A)symmetric dimethylarginine | ↑ | = | 0.07 |
| **BH4 metabolism** | Nicotinamide riboside | ↑ | = | 0.41 |
| | **Phenylalanyl-Asparagine / Asparaginyl-Phenylalanine** | ↑ | ↓ | **0.03*** |
| | **Metabolite 4** | ↑ | ↓ | **0.04*** |
| | **Dihydropteridine** | ↑ | ↓ | **0.004*** |
| **Anti-oxidants** | **Alpha-Tocotrienol** | ↑ | ↓ | **0.0002*** |
| | 9'-Carboxy-alpha-tocotrienol / 12a-Hydroxy-3-oxocholadienic acid | ↑ | = | 0.05 |
| | **9'-Carboxy-gamma-tocotrienol** | ↑ | ↓ | **0.02*** |
| | Alpha-CEHC / Monoethylhexyl phthalic acid | ↑ | ↑ | 0.008* |
| | **3-Hydroxymelatonin** | ↓ | ↑ | **0.01*** |
| **Homocysteine metabolism** | L-Homocysteine sulfonic acid | ↓ | = | 0.45 |
| | Cysteinyl-Alanine / Alanyl-Cysteine | ↓ | = | 0.08 |
| | 1-Methylhypoxanthine / 7-Methylhypoxanthine | ↓ | ↓ | 0.004* |
| | Diphthamide | ↑ | = | 0.15 |
| | **3-Polyprenyl-4,5-dihydroxybenzoate** | ↑ | ↓ | **<0.0001*** |
| **Eicosanoids** | Metabolite 1 | ↑ | ↑ | 0.04* |
| | Metabolite 3 | ↑ | ↑ | 0.03* |
| | **13,14-Dihydro PGE1 / 13,14-Dihydro PGF2a / Prostaglandin F1a** | ↑ | ↓ | **0.02*** |
| **Fatty acids** | trans-Dodec-2-enoic acid / 5-Dodecenoic acid | ↑ | ↑ | 0.04* |
| | LPA(16:0/0:0) / LPA(0:0/16:0) | ↑ | = | 0.28 |
| | 2,3-Methylene suberic acid / 3,4-Methylene suberic acid | ↑ | ↑ | 0.02* |
| | **Alpha-linolenyl carnitine / Gamma-linolenyl carnitine** | ↑ | ↓ | **0.03*** |
| | **14-HDoHE** | ↑ | ↓ | **0.007*** |
| **Steroids** | **Metabolite 2** | ↑ | ↓ | **<0.0001*** |
| | **Metabolite 5** | ↓ | ↑ | **0.006*** |
| | **24,25,26,27-Tetranor-23-oxo-hydroxyvitamin D3** | ↑ | ↓ | **0.01*** |
| | **11beta,20-Dihydroxy-3-oxopregn-4-en-21-oic acid** | ↑ | ↓ | **0.0004*** |
| **Unknown** | 4-Hydroxy-3-methoxy-cinnamoylglycine | ↑ | = | 0.08 |

P values from one-tailed paired t-tests between metabolites in AS patient sera taken before and 4 months after AVR.
*Significant difference (increase or decrease indicated by the arrows) at $p<0.05$. Unchanged (=) when $p>0.05$. Metabolites which show a significant reversal after AVR are indicated in bold.

**Supplemental Table 4.** Correlation between the top 30 metabolites of the metabolic profile and left ventricular mass (LVM) indexed for body surface area (BSA) and myocardial external efficiency indexed for BSA, respectively LVMi and MEEi.

| Metabolite | Before AVR | After AVR | LVMi | | | MEEi | | |
|---|---|---|---|---|---|---|---|---|
| | | | $R^2$ | P value | FDR | $R^2$ | P value | FDR |
| Homo-L-Arginine | ↑ | = | 0.62 | <0.0001 | 0.048204 | 0.49 | 0.0009 | 0.179834 |
| L-Arginine | ↑ | ↑ | 0.6 | <0.0001 | 0.048204 | 0.51 | 0.0006 | 0.155335 |
| (A)symmetric dimethylarginine | ↑ | = | 0.57 | 0.0002 | 0.069656 | 0.4 | 0.0035 | 0.343254 |
| Nicotinamide riboside | ↑ | = | 0.11 | 0.1677 | 0.77763 | 0.11 | 0.167 | 0.813088 |
| **Phenylalanyl-Asparagine / Asparaginyl-Phenylalanine** | **↑** | **↓** | **0.55** | **0.0003** | **0.070049** | **0.66** | **<0.0001** | **0.049699** |
| Metabolite 4 | ↑ | ↓ | 0.39 | 0.0043 | 0.230593 | 0.3 | 0.0152 | 0.569802 |
| Dihydropteridine | ↑ | ↓ | 0.37 | 0.0054 | 0.274244 | 0.28 | 0.0207 | 0.569802 |
| Alpha-Tocotrienol | ↑ | ↓ | 0.32 | 0.0119 | 0.461431 | 0.13 | 0.1224 | 0.765295 |
| 9'-Carboxy-alpha-tocotrienol / 12a-Hydroxy-3-oxocholadienic acid | ↑ | = | 0.53 | 0.0004 | 0.074217 | 0.28 | 0.021 | 0.569802 |
| **9'-Carboxy-gamma-tocotrienol** | **↑** | **↓** | **0.6** | **0.0001** | **0.048204** | **0.43** | **0.0025** | **0.306104** |
| Alpha-CEHC / Monoethylhexyl phthalic acid | ↑ | ↑ | 0.51 | 0.0006 | 0.092942 | 0.31 | 0.0126 | 0.569802 |
| 3-Hydroxymelatonin | ↓ | ↑ | 0.2 | 0.0524 | 0.666301 | 0.26 | 0.0252 | 0.604403 |
| L-Homocysteine sulfonic acid | ↓ | = | 0.15 | 0.0965 | 0.693427 | 0.18 | 0.0722 | 0.722006 |
| Cysteinyl-Alanine / Alanyl-Cysteine | ↓ | = | 0.37 | 0.0061 | 0.292895 | 0.32 | 0.0109 | 0.569802 |
| 1-Methylhypoxanthine / 7-Methylhypoxanthine | ↓ | ↓ | 0.2 | 0.0555 | 0.666301 | 0.33 | 0.0108 | 0.569802 |
| Diphthamide | ↑ | = | 0.23 | 0.0377 | 0.663081 | 0.23 | 0.0393 | 0.685654 |
| **3-Polyprenyl-4,5-dihydroxybenzoate** | **↑** | **↓** | **0.55** | **0.0003** | **0.070049** | **0.51** | **0.0006** | **0.155335** |
| trans-Dodec-2-enoic acid / 5-Dodecenoic acid | ↑ | ↑ | 0.54 | 0.0004 | 0.074217 | 0.499 | 0.0007 | 0.168447 |
| LPA(16:0/0:0) / LPA(0:0/16:0) | ↑ | = | 0.45 | 0.0016 | 0.143242 | 0.25 | 0.0284 | 0.645321 |
| 2,3-Methylene suberic acid / 3,4-Methylene suberic acid | ↑ | ↑ | 0.31 | 0.0126 | 0.477657 | 0.39 | 0.0041 | 0.379805 |
| Alpha-linolenyl carnitine / Gamma-linolenyl carnitine | ↑ | ↓ | 0.21 | 0.0501 | 0.666301 | 0.17 | 0.0841 | 0.722006 |
| 14-HDoHE | ↑ | ↓ | 0.47 | 0.0013 | 0.131652 | 0.29 | 0.0184 | 0.569802 |
| Metabolite 1 | ↑ | ↑ | 0.27 | 0.0239 | 0.604347 | 0.24 | 0.032 | 0.650984 |
| Metabolite 2 | ↑ | ↑ | 0.41 | 0.0031 | 0.292895 | 0.3 | 0.0158 | 0.569802 |
| 13,14-Dihydro PGE1 / 13,14-Dihydro PGF2a / Prostaglandin F1a | ↑ | ↓ | 0.22 | 0.0419 | 0.663081 | 0.2 | 0.0537 | 0.700883 |
| Metabolite 3 | ↑ | ↓ | 0.36 | 0.0064 | 0.201124 | 0.29 | 0.0179 | 0.569802 |
| Metabolite 5 | ↓ | ↑ | 0.15 | 0.0976 | 0.693427 | 0.23 | 0.0373 | 0.685654 |
| 24,25,26,27-Tetranor-23-oxo-hydroxyvitamin D3 | ↑ | ↓ | 0.49 | 0.0008 | 0.100121 | 0.25 | 0.031 | 0.650984 |
| 11beta,20-Dihydroxy-3-oxopregn-4-en-21-oic acid | ↑ | ↓ | 0.496 | 0.0008 | 0.100121 | 0.41 | 0.0033 | 0.342398 |
| 4-Hydroxy-3-methoxy-cinnamoylglycine | ↑ | = | 0.4 | 0.0037 | 0.212982 | 0.48 | 0.0011 | 0.198779 |

A linear regression analysis was performed with Pearson correlation and p-values were adjusted for False Discovery Rate (FDR) using the Benjami-Hochberg correction.
For overview purposes, the arrows indicate whether a metabolite is increased or decreased compared to controls (before AVR) and compared to after AVR. The colours correspond to the categories of metabolites shown in figures 3 through 11.

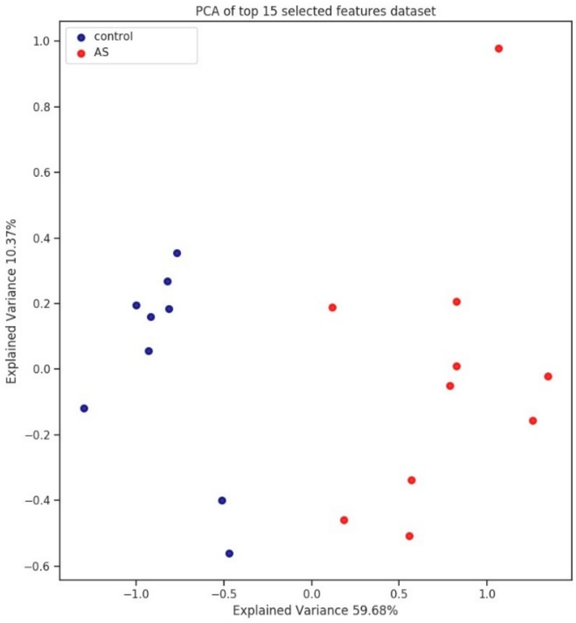


**Figure S1.** Principal component analysis (PCA) plot shows good separation of the model between controls and AS patients.

Figure S2.

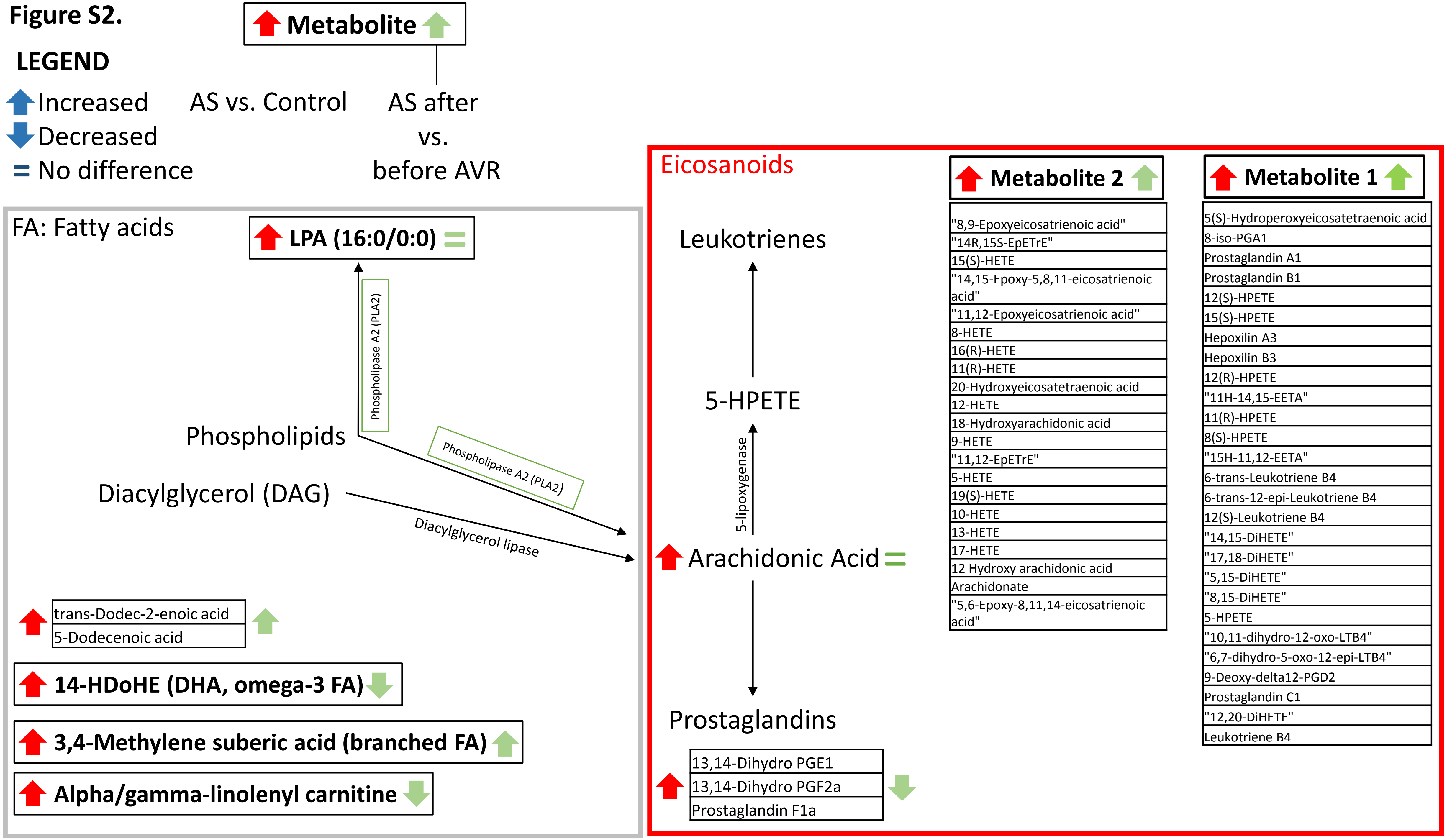

Metabolite
LEGEND
Increased
Decreased
= No difference
AS vs. Control
AS after
vs.
before AVR
FA: Fatty acids
LPA (16:0/0:0)
Phospholipase A2 (PLA2)
Phospholipids
Phospholipase A2 (PLA2)
Diacylglycerol (DAG)
Diacylglycerol lipase
trans-Dodec-2-enoic acid
5-Dodecenoic acid
14-HDoHE (DHA, omega-3 FA)
3,4-Methylene suberic acid (branched FA)
Alpha/gamma-linolenyl carnitine
Eicosanoids
Leukotrienes
5-HPETE
5-lipoxygenase
Arachidonic Acid
Prostaglandins
13,14-Dihydro PGE1
13,14-Dihydro PGF2a
Prostaglandin F1a
Metabolite 2
"8,9-Epoxyeicosatrienoic acid"
"14R,15S-EpETrE"
15(S)-HETE
"14,15-Epoxy-5,8,11-eicosatrienoic acid"
"11,12-Epoxyeicosatrienoic acid"
8-HETE
16(R)-HETE
11(R)-HETE
20-Hydroxyeicosatetraenoic acid
12-HETE
18-Hydroxyarachidonic acid
9-HETE
"11,12-EpETrE"
5-HETE
19(S)-HETE
10-HETE
13-HETE
17-HETE
12 Hydroxy arachidonic acid
Arachidonate
"5,6-Epoxy-8,11,14-eicosatrienoic acid"
Metabolite 1
5(S)-Hydroperoxyeicosatetraenoic acid
8-iso-PGA1
Prostaglandin A1
Prostaglandin B1
12(S)-HPETE
15(S)-HPETE
Hepoxilin A3
Hepoxilin B3
12(R)-HPETE
"11H-14,15-EETA"
11(R)-HPETE
8(S)-HPETE
"15H-11,12-EETA"
6-trans-Leukotriene B4
6-trans-12-epi-Leukotriene B4
12(S)-Leukotriene B4
"14,15-DiHETE"
"17,18-DiHETE"
"5,15-DiHETE"
"8,15-DiHETE"
5-HPETE
"10,11-dihydro-12-oxo-LTB4"
"6,7-dihydro-5-oxo-12-epi-LTB4"
9-Deoxy-delta12-PGD2
Prostaglandin C1
"12,20-DiHETE"
Leukotriene B4

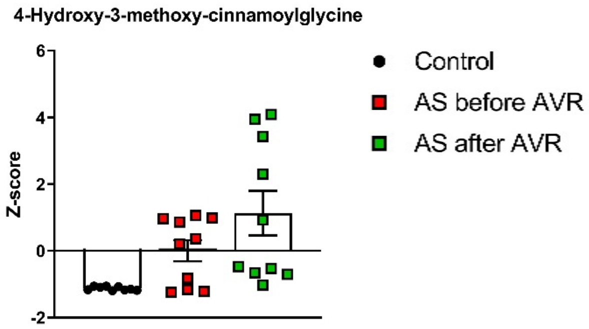


**Figure S3.** 4-hydroxy-3-methoxy-cinnamoylglycine is of unknown biological relevance.
